# Solitary Fibrous Tumor of Pleura Invading into the Left Atrium via Pulmonary Vein

**DOI:** 10.7759/cureus.3473

**Published:** 2018-10-22

**Authors:** Arsalan Talib Hashmi, Sushilkumar S Gupta, Arjun Saradna, Asiya Batool, Abhinav Saxena, Stephan Kamholz

**Affiliations:** 1 Internal Medicine, Maimonides Medical Center, Brooklyn, USA; 2 Critical Care, Maimonides Medical Center, Brooklyn, USA; 3 Internal Medicine, Jinnah Hospital Lahore (JHL)/Allama Iqbal Medical College (AIMC), Lahore , PAK; 4 Cardiology, Maimonides Medical Center, Brooklyn, USA; 5 Pulmonology, Maimonides Medical Center, Brooklyn, USA

**Keywords:** hemangiopericytoma, solitary fibrous tumor, mesenchymal tumor

## Abstract

A 67-year-old woman came to the hospital because of difficulty in breathing. After an initial clinical assessment, contrast-enhanced computerized tomography (CT) of the chest revealed a well-circumscribed heterogeneous mass arising from the pleura adjacent to the superior and medial left pulmonary artery. The mass was invading the pulmonary vein and entering the left atrium. Histopathology of the biopsy of the mass was suggestive of solitary fibrous tumor (SFT) of the pleura. The patient underwent pneumonectomy and resection of the left atrial mass with pericardial patch repair of the left atrium.

## Introduction

Solitary fibrous tumors (SFTs) are mesenchymal in origin, rarely metastasize and include hemangiopericytoma. Hemangiopericytoma is now preferably termed SFT by most pathologists [1]. Solitary fibrous tumor of pleura (SFTP) is an exceedingly rare tumor of pleura. We describe a woman with the recurrence of SFTP who had a previous history of hemangiopericytoma of the neck treated several years ago.

## Case presentation

A 67-year-old woman presented to the emergency department with difficulty in breathing. She had a past medical history of hypertension, hyperlipidemia, and hemangiopericytoma of the neck treated by surgical resection and radiation therapy 20 years ago. At the hospital, her vital signs were stable with a heart rate of 96 beats per minute, respiratory rate of 20 per minute, blood pressure of 138/78 mmHg, and percentage oxygen saturation of 96% on room air. Review of systems was otherwise unremarkable. The cardiovascular, respiratory, and abdominal examination did not reveal any pathological findings. Complete blood count showed mild anemia with a hemoglobin level of 11 g/dL. The basic metabolic panel, venous blood gas, and liver profile were unremarkable.

**Figure 1:**
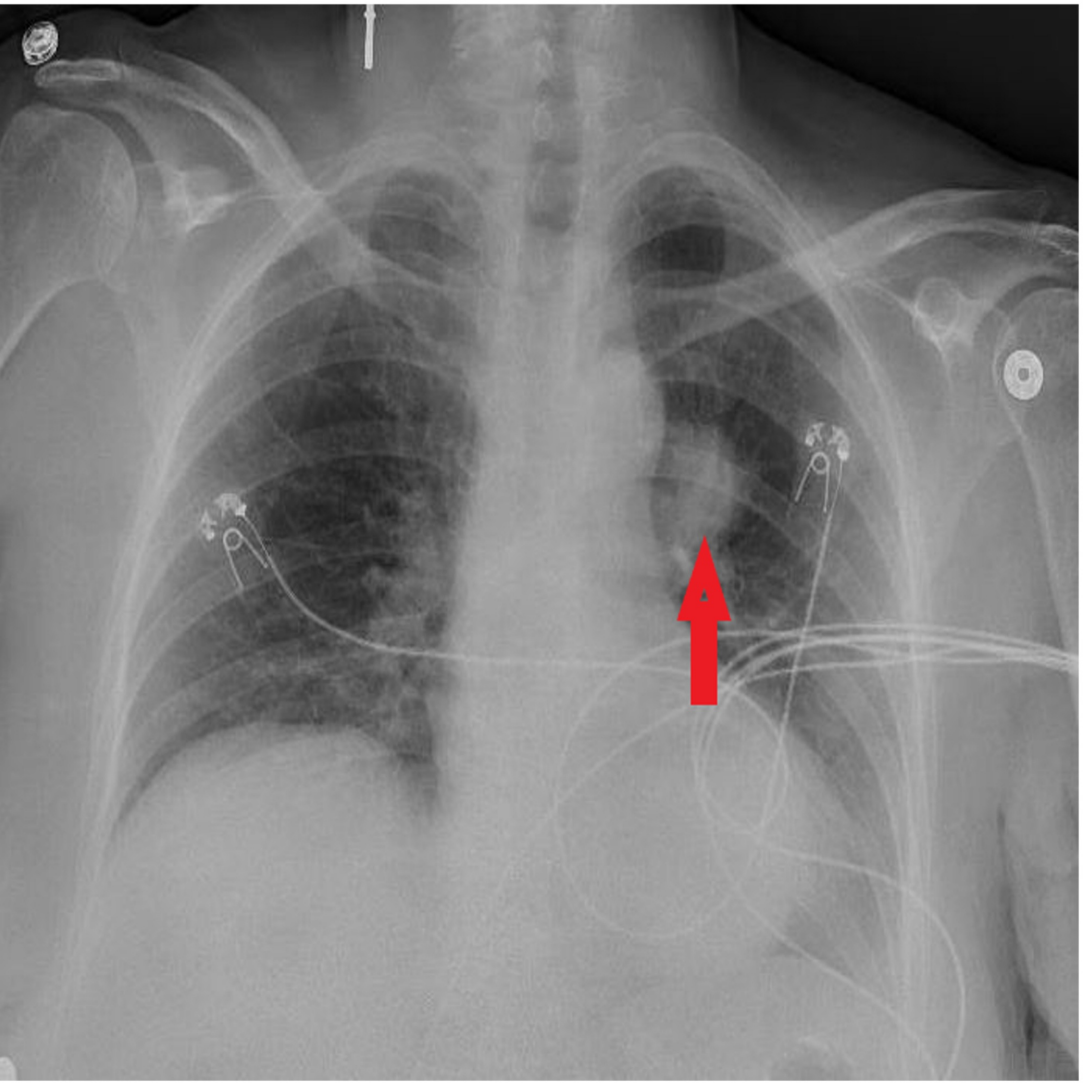
Chest X-ray showing round mass-like opacity in left suprahilar region

Chest X-ray showed a round mass-like opacity in the left suprahilar region (Figure 1).

**Figure 2:**
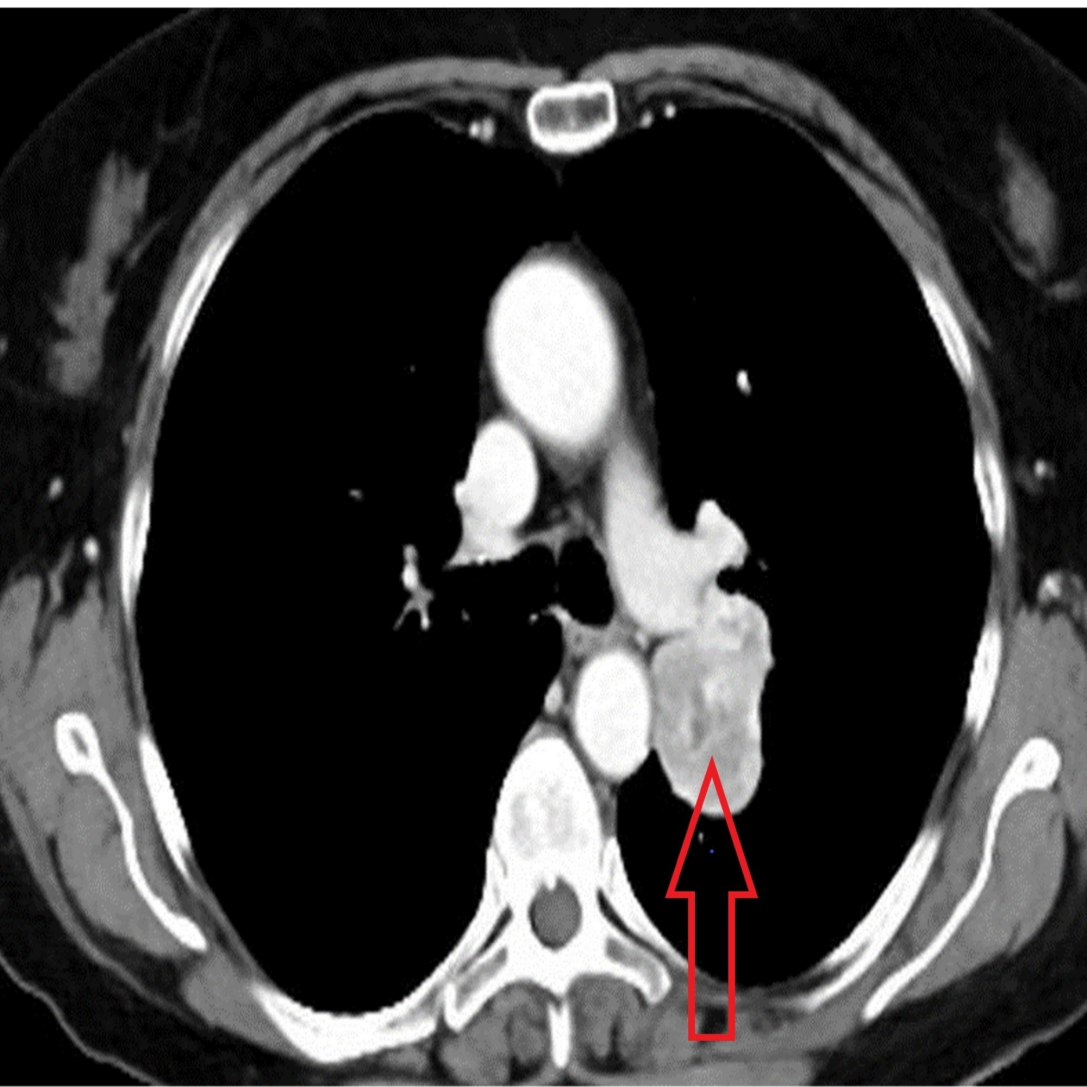
Heterogeneous mass arising from the pleura adjacent to the superior and medial left pulmonary artery

Computerized tomography (CT) scan of the chest with intravenous contrast enhancement revealed a well-circumscribed heterogeneous mass arising from the pleura adjacent to the superior and medial left pulmonary artery (Figure 2). 

**Figure 3:**
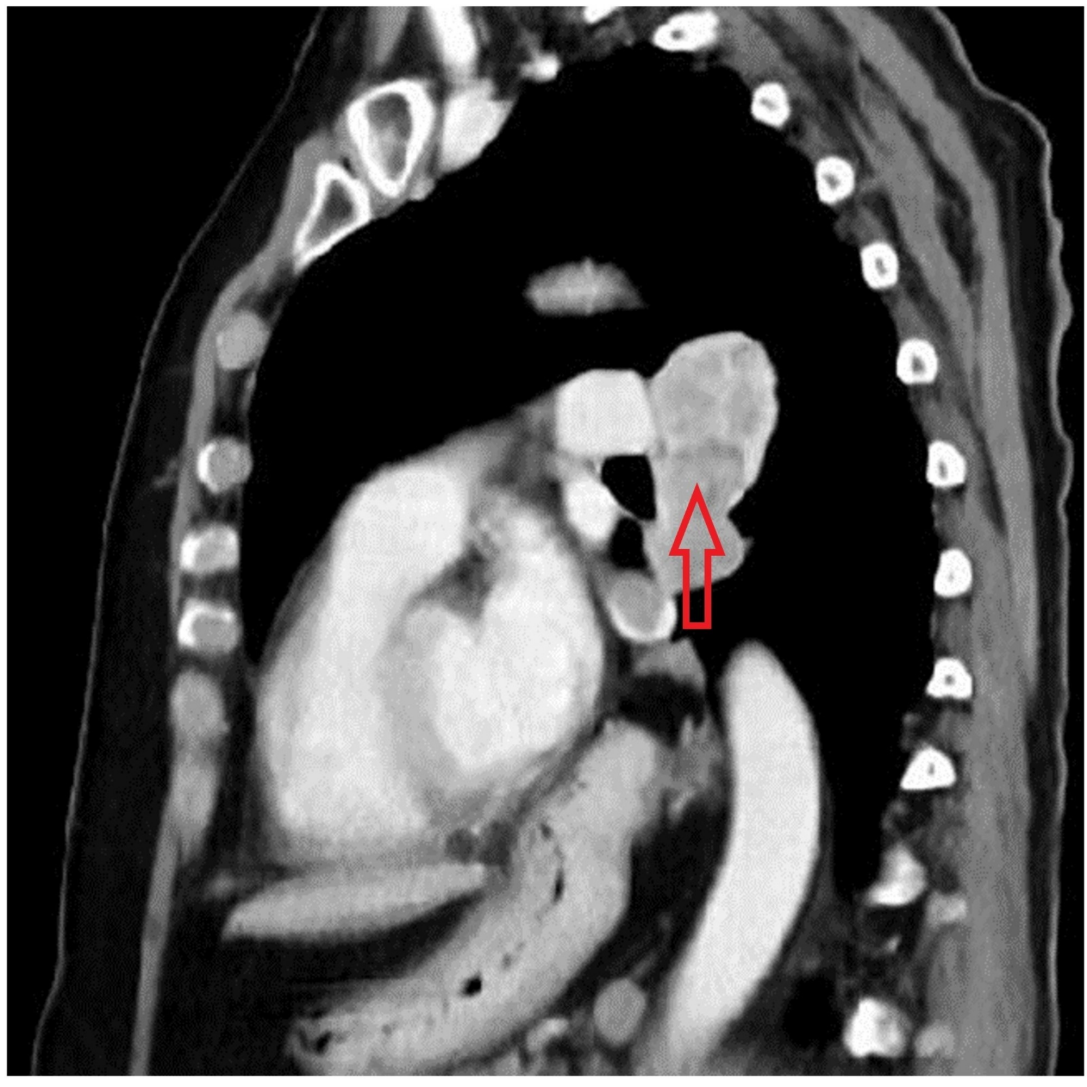
Invasion of the left inferior pulmonary vein with extension into the left atrium

At the inferior extent of the tumor, there was an invasion of the left inferior pulmonary vein with extension into the left atrium (Figure 3).

**Figure 4:**
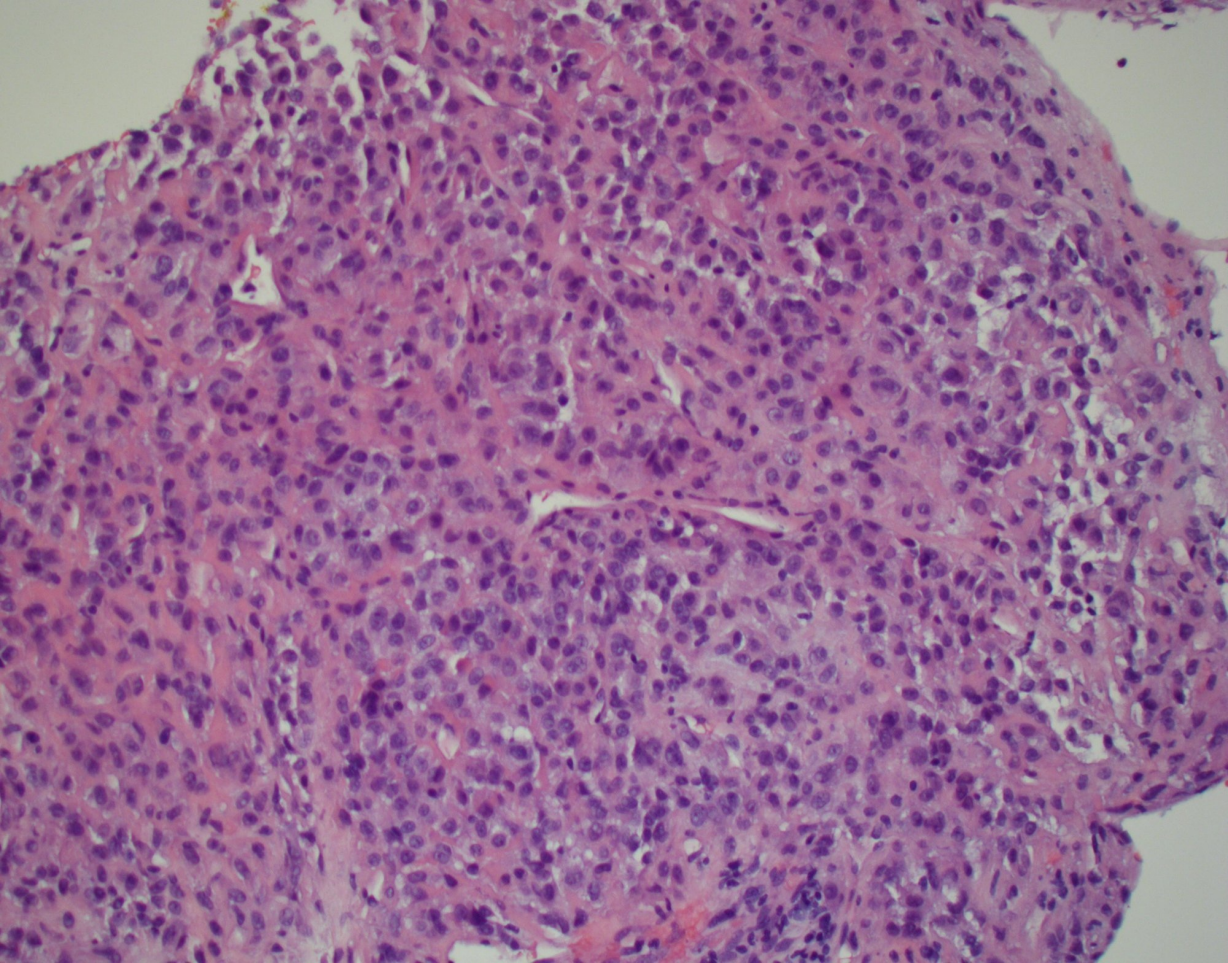
Light microscopy of the biopsied specimen showing spindle and epithelioid cell neoplasm

The lesion was biopsied confirming a mesenchymal neoplasm compatible with SFT (Figure 4).

The patient underwent pneumonectomy and resection of left atrial mass with pericardial patch repair of the left atrium. Postoperatively, her hospital course was complicated with left-sided pneumothorax requiring chest tube placement, septic shock requiring vasopressors, acute respiratory distress syndrome (ARDS) requiring mechanical ventilation and acute renal failure requiring hemodialysis. She underwent tracheostomy and percutaneous gastrostomy tube placement. Despite efforts of the multidisciplinary team, the patient did not show clinical improvement. The family then decided to do not resuscitate (DNR) in case of cardiac arrest. Unfortunately, due to above-stated complications, the patient died in the hospital on day 107.

## Discussion

Prior terminology for “solitary fibrous tumor (SFT)" has included pleural mesothelioma, localized fibrous mesothelioma, and localized fibrous tumor. Our patient had a previous history of hemangiopericytoma of the neck, now referred to as SFT [1].

The localized fibrous tumor can occur anywhere including soft tissue and viscera, with a preference for serosal membranes including the pleura, peritoneum, and meninges. The first histopathologic description of SFTP was published by Klemperer and Rabin in 1931. They distinguished localized mesotheliomas from diffuse mesothelioma and proposed that submesothelial cells were the cellular origin for a localized variety [2].

SFTP is very rare, representing only 5% of all pleural tumors. Only 800 cases were reported in the literature between 1931 and 2002 [3]. SFTP usually present in older patients (sixth to the seventh decade), with similar frequency in men and women [4]. Cough, chest pain and dyspnea are the most common presenting symptoms. There are no known environmental, inherited or predisposing risk factors. Clubbing, hypertrophic osteoarthropathy, and hypoglycemia are paraneoplastic associations. Hypertrophic osteoarthropathy is thought to be due to abnormal production of hyaluronic acid, while hypoglycemia is believed to be due to the production of insulin-like growth factor 2 (IGF2) [5]. Radiographically, both benign and malignant variants usually appear well circumscribed. SFTP usually appear well-delineated, and are often lobulated, with heterogeneous attenuation on CT scan [6]. The majority of SFTP originate from the visceral pleura and are pedunculated, as in our patient.

## Conclusions

SFTP have a very low risk of recurrence or metastasis. Surgical resection is the mainstay of treatment with good prognosis in most patients. Although usually benign, occasionally SFTP may enlarge rapidly and transform into malignant variants. Therefore, complete surgical resection and careful long-term follow-up are recommended for all patients.
